# Exercise and NO production: relevance and implications in the cardiopulmonary system

**DOI:** 10.3389/fcell.2014.00073

**Published:** 2015-01-07

**Authors:** Alexei V. Nosarev, Lyudmila V. Smagliy, Yana Anfinogenova, Sergey V. Popov, Leonid V. Kapilevich

**Affiliations:** ^1^Institute of Physics and Technology, National Research Tomsk Polytechnic UniversityTomsk, Russia; ^2^Department of Biophysics and Functional Diagnostics, Siberian State Medical UniversityTomsk, Russia; ^3^Research Institute for Cardiology, Federal State Budgetary Scientific InstitutionTomsk, Russia; ^4^Faculty of Physical Education, National Research Tomsk State UniversityTomsk, Russia

**Keywords:** nitric oxide, nitric oxide synthase, exercise, cardiopulmonary system, blood vessels

## Abstract

This article reviews the existing knowledge about the effects of physical exercise on nitric oxide (NO) production in the cardiopulmonary system. The authors review the sources of NO in the cardiopulmonary system; involvement of three forms of NO synthases (eNOS, nNOS, and iNOS) in exercise physiology; exercise-induced modulation of NO and/or NOS in physiological and pathophysiological conditions in human subjects and animal models in the absence and presence of pharmacological modulators; and significance of exercise-induced NO production in health and disease. The authors suggest that physical activity significantly improves functioning of the cardiovascular system through an increase in NO bioavailability, potentiation of antioxidant defense, and decrease in the expression of reactive oxygen species-forming enzymes. Regular physical exercises are considered a useful approach to treat cardiovascular diseases. Future studies should focus on detailed identification of (i) the exercise-mediated mechanisms of NO exchange; (ii) optimal exercise approaches to improve cardiovascular function in health and disease; and (iii) physical effort thresholds.

## Introduction

Nitric oxide (NO) is a free radical with the high reactivity and diffusion rate (Garthwaite et al., 1988; Archer, 1993; Brunori et al., 2006). Direct measurements of NO content in biological tissues are complicated due to NO binding to hemoglobin (Lundberg et al., 1994; Griffiths et al., 2003; Brunori et al., 2006). At the same time, NO is stable at the low concentrations ranging from 0.1 to 100 nM (Archer, 1993; Griffiths et al., 2003). Due to this, NO, synthesized in the hollow organs, diffuses into the lumen where it can be detected in the gaseous phase (Archer, 1993). Nitric oxide, synthesized in the airways, can be detected in the exhaled air (Alving et al., 1993; Lundberg et al., 1994). Nitric oxide content in the exhaled air can characterize metabolic (Whittle, 1995) and physiological conditions of the respiratory organs (Alving et al., 1993; Barnes and Kharitonov, 1996; Dweik et al., 1998).

Nitric oxide is produced by various cells (Archer, 1993; Bauer et al., 1994, p. 62). Numerous studies suggest that NO synthesis depends on physical stimuli that modulate the activity of NO synthases (NOS) (Laughlin et al., 2001; Gielen et al., 2005; Park et al., 2012). Three isoforms of NOS were identified: neuronal (nNOS), macrophage or inducible (iNOS), and endothelial (eNOS) isoforms encoded by distinct genes (Ricciardolo, 2003; Garcia, 2011).

Available literature presents controversial data on exercise-induced changes in eNOS expression.

There is evidence that 24-week course of swimming exercise does not change expression of eNOS protein in healthy mice (Pellegrin et al., 2011). On other hand, swimming exercise increases eNOS expression at the protein level in mice prone to hypercholesterolemia and atherosclerosis (Pellegrin et al., 2007, 2011). Moreover, eNOS protein expression and phosphorylation is increased in porcine coronary arteries in the models of chronic coronary occlusion and stenosis (Heaps et al., 2006). Coronary artery disease (CAD) patients, subjected to bicycle ergometer exercise, have twice higher levels of eNOS protein expression and phosphorylation compared with CAD patients with sedentary lifestyle (Hambrecht et al., 2003; Gielen et al., 2010). Other data demonstrate that exercise stimulates endothelium-dependent relaxation of collateral coronary arteries and arterioles in healthy animals. This effect is associated with increases in eNOS expression at the mRNA and protein levels (Laughlin et al., 2001). Exercise effects on vascular endothelium are mediated by stepwise increase in shear stress. An increase in the shear stress is caused by elevation of cardiac output during physical exercise (Persson et al., 1993). Signaling in the endothelial surface, exposed to the vascular lumen, is triggered by deformation of glycocalyx (Reitsma et al., 2007).

Exercise does not significantly affect nNOS in spontaneously hypertensive rats (Park et al., 2012). In patients with chronic heart failure, exercise decreases the expression of cytokines and iNOS in the muscular biopsies (Kingwell, 2000; Boo and Jo, 2003). The effects of physical exercise on iNOS and nNOS are still poorly understood. Many questions on how exercise modulates the activity of NOS isoforms remain unresolved.

### Sources of nitric oxide in cardiopulmonary system

**Figure 1:**
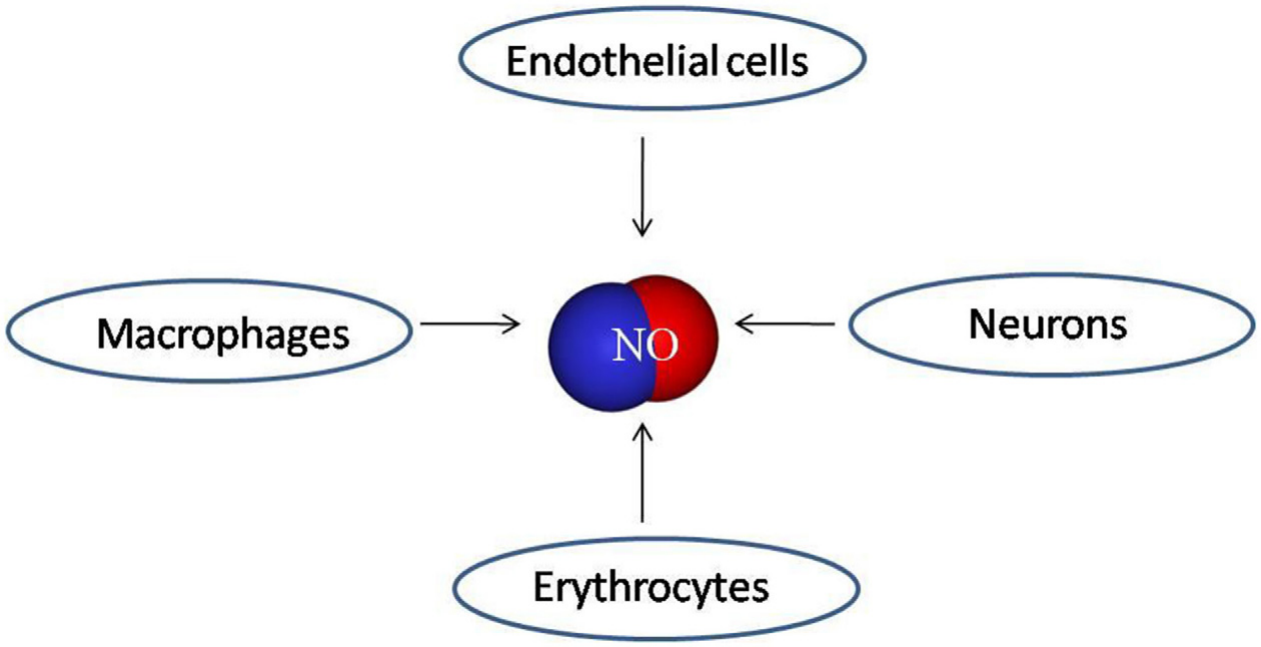
**Main cell types involved in NO production in the cardiopulmonary system**.

Nitric oxide is detected in the exhaled air (Alving et al., 1993; Lundberg et al., 1994). However, the sources of NO in the exhaled air have not been clearly identified. Nitric oxide can be produced by various cells present in the lungs (Spriestersbach et al., 1995) including epithelial, neural, inflammatory (macrophages, neutrophils, and mast cells) (Gaston et al., 1994), and vascular endothelial cells (Ignarro et al., 1987; Garthwaite, 2008) (Figure 1). Significant portion of the exhaled NO is produced by the endothelium of the microvessels and pulmonary blood vessels and alveolar epithelium (Kharitonov et al., 1996; Dweik et al., 1998).

Nitric oxide can be also transported to the lungs by blood flow (Gaston et al., 1994). After release of NO into the blood stream, NO binds to hemoglobin and then it is delivered to the alveoli (Lundberg et al., 1994; Green et al., 2011). Due to a high diffusion ability (diffusion coefficient is approximately 3300 μm^2^/s), NO easily diffuses into the gaseous phase of the lungs (Ricciardolo, 2003).

The exhaled NO level is lower in patients with coronary insufficiency compared with healthy individuals (Sumino et al., 1998; Clini et al., 2000). Exercise stimulates NO release from the endothelial cells (Moncada, 1997). Regular exercises improve functioning of the blood vessels and contribute to the better myocardial perfusion and contractile function in subjects with vascular diseases (Hambrecht et al., 2000, 2003; Xie et al., 2012). However, despite obvious benefits of physical training, the main exercise-mediated mechanisms involved in the improvement of the vascular function in coronary artery disease have not been clearly identified.

Nitric oxide exchange occurs in the alveoli and other parts of the airways and significantly depends on the exhaled air flow (Silkoff et al., 1997; Tsoukias et al., 1998). The latter complicates the interpretation of NO oscillations in the exhaled air in various clinical and physiological conditions (Tsoukias and George, 1998; Shin et al., 2001, 2002; Tsoukias et al., 2001).

Nitric oxide diffuses from the endothelial cells into the vascular smooth muscle cells where this molecule activates guanylate cyclase producing cyclic guanosine-3,5-monophosphate (cGMP) from GTP. cGMP is a second messenger whose important effect consists in the relaxation of the blood vessels (Vallance and Chan, 2001). Liao et al. (1999) studied the processes of endothelial NO diffusion into the adjacent smooth muscle cells of the porcine pulmonary blood vessels. These researchers hypothesized that hemoglobin binds almost all available NO. They also showed that erythrocytes do not disrupt NO-mediated vasodilation in the isolated microvessels when the intravascular blood flow is preserved. Therefore, the erythrocytes are not scavengers of NO in the given conditions (Liao et al., 1999). Vaughn et al. (2000) demonstrated that limited transmembrane diffusion decreases NO absorption by the erythrocytes.

At the same time, the erythrocytes contribute to the NO-mediated vasodilation in the absence of intravascular blood flow. Kleinbongard et al. (2006) provided evidence of eNOS presence in human and murine erythrocytes suggesting a possibility for NO syntheses from L-arginine by the erythrocytes. Possible sources of vasoactive NO comprise S-nitrosohemoglobin (SNOHb) and nitrite. However, there is a lack of convincing data on how NO or its equivalents are transported to a vascular wall from either source: SNOHb or nitrites (Robinson and Lancaster, 2005). Nitric oxide can be directly released from the erythrocytes into the extracellular space or be formed outside erythrocytes from an intermediate product generated during reaction between nitrite and deoxyhemoglobin (nitrite-deoxyHb reaction) (Kim-Shapiro et al., 2005; Crawford et al., 2006). Studies demonstrated that redox-active thiols, abundant in blood plasma, can bind NO and transport it in the form of bioactive S-nitrosothiols (RSNOs) in the bloodstream (Stamler et al., 1992a). In the presence of oxygen, S-nitroso-albumin (SNOAlb) is considered to be main product of NO binding (Stamler et al., 1992b; Marley et al., 2001). However, mechanisms of formation and release of NO from SNOAlb and other RSNOs *in vivo* remain completely unclear (Rassaf et al, 2002).

### Nitric oxide synthases

#### eNOS

Endothelial NO synthase (eNOS) is a membrane-bound isoform of the enzyme localized in the caveolae, small invaginations of plasma membrane containing transmembrane protein caveolin (Ricciardolo, 2003; Förstermann and Sessa, 2012). eNOS is found in the lungs, trachea (Zhan et al., 2003), alveolar and bronchial epithelial cells (Pechkovsky et al, 2002), alveolar macrophages (Shaul et al., 1994; Giaid and Saleh, 1995; Aminuddin et al., 2013), vascular smooth muscle cells (Zhan et al., 2003), pulmonary endothelium, and endothelial cells of the blood vessels feeding the airways (Curzen et al., 1996; Patel et al., 1999). Immunohistochemistry approaches enabled researchers to show that eNOS is localized in the respiratory ciliated epithelium. This enzyme is present in the basal bodies of the cilia and increases the ciliary beat frequency (Zhan et al., 2003). eNOS is a calcium-dependent isoform producing discrete NO quanta (Patel et al., 1999). The activity of eNOS is suppressed when the enzyme binds to caveolin in the endothelial cells. In the presence of the agonist-induced Ca^2+^ currents, eNOS binds to calmodulin and dissociates from caveolin. Synthesis of NO by the complex of eNOS-calmodulin continues until the Ca^2+^ currents have decreased and the inhibitory eNOS-caveolin complex has formed (Michel and Feron, 1997). Nitric oxide, synthesized by eNOS, is involved in the regulation of arterial blood pressure and airway lumen diameter (Whittle, 1995).

### nNOS

Neuronal NO synthase (nNOS) is a soluble cytosolic isoform of NO synthase (NOS) (Ricciardolo, 2003). nNOS is found in different cells present in the lungs including neuronal (Tzao et al., 2000; Lührs et al., 2002), epithelial (Belvisi et al., 1995), and endothelial cells (Shaul et al., 1994; Aminuddin et al., 2013). The presence of nNOS in the nerve fibers is demonstrated by immunohistochemistry and NADPH-diaphoresis histochemistry (Fischer et al., 2002).

In the peripheral blood vessels of the lungs, smooth muscles are innervated by the NO-ergic nerves, i.e., the nerves that express nNOS and therefore generate and release NO. Nitric oxide, produced by nNOS in the NO-ergic nerves, can be considered as a neurotransmitter stimulating NO-sensitive guanylate cyclase in different types of smooth muscle cells of the blood vessels and airways (Ward et al., 1995; Patel et al., 1999; Förstermann and Sessa, 2012). Similarly to eNOS, nNOS requires calcium ions to produce NO (Whittle, 1995).

#### iNOS

According to current knowledge, inducible NO synthase (iNOS) is present in the macrophages and other cells present in the lungs (Fischer et al., 2002). In particular, iNOS is expressed in the epithelial cells of human airways (Guo et al., 1995; Pechkovsky et al, 2002), type II alveolar epithelium, pulmonary endothelium (Aminuddin et al., 2013), lung fibroblasts (Romanska et al., 2002), bronchial and vascular smooth muscle cells (Xue and Johns, 1996), must cells (Gilchrist et al., 2002), endothelial cells (Ermert et al., 2002), chondrocytes, and neutrophils (Blackford et al., 1994). Similarly to nNOS, iNOS is a soluble cytosolic protein (Ricciardolo, 2003).

Unlike eNOS and nNOS, iNOS is Ca^2+^-independent enzyme that generates NO more abundantly (nanomolar and micromolar concentrations) compared with other NOS isoforms and maintains NO production for hours and days (Ricciardolo, 2003). A large number of studies (Pautz et al., 2010; Cortese-Krott et al., 2014) demonstrated that iNOS can produce nitric oxide in micromolar concentrations. These high levels of iNOS-derived nitric oxide have been shown to be involved in pathological conditions, e.g., the blood pressure fall in septic shock as well as in the pathogenesis of chronic inflammatory diseases, including atherosclerosis. Inducible NOS is controlled at the pretranslational level. iNOS upregulation is triggered by the proinflammatory cytokines: tumor necrosis factor (TNF)-α, interferon (INF)-γ, and interleukin (IL)-1β (Morris and Billiar, 1994). Increase in the production of NO by iNOS is observed in response to the endotoxins (Michel and Feron, 1997). Activation of iNOS is triggered by the pathophysiological events associated with inflammation (Whyte and Laughlin, 2010). Cytokine-induced stimulation activates iNOS in different cell types (Ito et al., 2013) except human bronchial smooth muscle cells (Förstermann et al., 1994; Patel et al., 1999; Maarsingh et al., 2009). High expression of iNOS in the presence of inflammation is documented in the murine bronchial smooth muscle cells (Kane et al., 1994; Maarsingh et al., 2009).

### Exercise-induced modulation of NO production

The extensive evidence points to the fact that exercise stimulates NO release. This explains why regular physical activity can slow down, suppress, or even reverse cardiovascular diseases (Alving et al., 1993; Carrizzo et al., 2013). However, literature also presents contradicting data suggesting that both NOS activity and NO production decrease in response to exercise (Miyauchi et al., 2003).

## Exercise-induced modulation of NO production in animal studies

Exercise modulates both the activity of eNOS and the expression of this enzyme at the mRNA and protein levels in the cells of aorta, heart, lung, and vena cava (Dao et al., 2011).

*In vivo* studies demonstrated the presence of exercise-mediated activation of Ca^2+^-dependent eNOS in murine lungs, aorta, and atria. Moreover, eNOS expression increases in the cells of cardiopulmonary system in laboratory animals (Tatchum-Talom et al., 2000). Physiological adaptation to swimming exercise potentiates acetylcholine-induced relaxation of blood vessels and nNOS activation in the endothelial cells of the lungs, atria, and aorta (Tatchum-Talom et al., 2000).

Many studies demonstrated that NO synthesis in the endothelial cells largely depends on the level of the individual physical activity. For example, exercise-induced relaxation of the collateral coronary arteries is associated with the increased expression of eNOS mRNA and protein in healthy animals (Sessa et al., 1994; Laughlin et al., 2001). Expression of eNOS mRNA is significantly higher in the lungs of animals subjected to physical exercise compared with resting animals. Western blot analysis demonstrated that eNOS is downregulated whereas iNOS is unchanged in the pulmonary tissue after exercise (Miyauchi et al., 2003). In porcine coronary arteries, the levels of both unphosphorylated and phosphorylated (Ser1179) eNOS increase after exercise in the model of chronic coronary occlusion/stenosis (Heaps et al., 2006). Hambrecht and coworkers demonstrated healing effects of exercise in patients with coronary artery disease. The study suggested that these beneficial effects are mediated through an increase in iNOS phosphorylation by protein kinase Akt (protein kinase B) (Hambrecht et al., 2003). Akt-kinase and AMP-activated protein kinase play a pivotal role in the phosphorylation of arterial eNOS at Ser1177 residue during running exercise (Zhang et al., 2009). Moien-Afshari et al. (2008) showed that seven-week physical training increases the level of eNOS phosphorylation in the cells derived from aorta of wild-type and diabetic rats (Patel et al., 1999). The increase in eNOS phosphorylation is considered to be an important molecular mechanism of adaptation to physical exercises (Shaul et al., 1994).

Data demonstrated that physical exercise decreases expression of iNOS at the mRNA and protein levels in the cells of blood vessels (Gielen et al., 2005). There is evidence that an increase in iNOS expression can occur under the influence of Toll-like receptors type 4 (TLR-4) through the activation of nuclear transcription factor-kappa B (NF-kB). The levels of TLR-4 and iNOS mRNAs increase after physical activity. Therefore, TLR-4 activation, mediated by NF-kB-dependent pathway, triggers the mechanisms of NO synthesis that can negatively affect myocardium in case of strenuous physical activity (Cristi-Montero et al., 2012).

## Impact of pharmacological modulation during physical efforts in training

Angelis with coworkers studied rats with normal blood pressure and hypertension caused by NOS blockage with N(omega)-nitro-L-arginine methyl ester. The study showed that exercise increases arterial blood pressure, heart rate, and cardiac output in normotensive rats. In hypertensive animals, physical exercise increases heart rate without affecting cardiac output, arterial blood pressure, and blood flow and is associated with a significant increase in arteriovenous oxygen gradient. Therefore, hypertension, associated with abnormal NO production, induces different cardiovascular adjustments to exercise (De Angelis et al., 2006).

In ovine pulmonary circulation system, vascular tone increases in response to α- and β-adrenergic stimulation present in exercise. The increase in NO production contributes to a significant decrease in α-adrenergic constriction of the pulmonary arteries during physical activity and dilates pulmonary blood vessels at rest (Kane et al., 1994). Experiments on sheep, subjected to physical exercise, demonstrated that intravenous infusion of L-arginine, a substrate for NO synthesis, reverses the effect of NO-synthase inhibition without affecting the tone of the pulmonary blood vessels (Koizumi et al., 1994). Similar responses to moderate/heavy exercise can be found in humans with preserved endothelial function (Green et al., 2011).

Exercise affects endothelium-independent relaxation in response to NO donor sodium nitroprusside. Therefore, NO-stimulated cGMP/PKG cascade in the smooth muscle cells of arterioles is not disrupted by physical exercise (Thengchaisri et al., 2007).

## Human studies

A vast pool of data suggests that exercise modulates NO synthesis in various tissues through altering NOS activity (Laughlin et al., 2001; Boo and Jo, 2003; Gielen et al., 2005; Park et al., 2012). Nitric oxide clearly affects physiological functions in exercise (Sheel et al., 1999). However, phenomenology and mechanisms of the exercise-induced effects on NO production remain largely unclear. Biological sample acquisition from humans subjected to physical exercise is challenging. In human studies, the changes in NO production are predominantly evaluated based on the measurements of NO content in the exhaled air.

Changes in NO content in the exhaled air have been demonstrated in many studies. Majority of the studies revealed a decrease in NO content in the exhaled air after physical exercise (Maroun et al., 1995, p. 102). However, available literature also presents data suggesting that physical training is associated with an increase in NO content in the exhaled air (Bauer et al., 1994; Bonsignore et al., 2001) or its invariance (Iwamoto et al., 1994; Maroun et al., 1995). This controversy is unsurprising due to the complexity of NO exchange and multisystemic nature of the physiological responses to physical exercise.

The concentration of NO in the exhaled air significantly decreases after exercise (Kippelen et al., 2002; Mantione et al., 2007). After training workout, NO levels in the exhaled air are significantly lower in the subjects who continue moving compared with those fully resting. These data suggest that NO content variations in the exhaled air depend on the individual levels of physical activity (Mantione et al., 2007).

Data show a small decrease in NO content in the exhaled air 3 min after exercise (St Croix et al., 1999). Measurements of NO in trained athletes before and after workout demonstrate a decrease in NO content in the exhaled air after exercise (Verges et al., 2005, 2006). At the same time, strenuous exercise potentiates NO diffusion from the pulmonary tissue to the gaseous phase (Shin et al., 2003).

Data suggest that NO synthesis in the pulmonary tissue depends on the oxygen content (Mantione et al., 2007). Decrease in NO concentration in the exhaled air suggests high NO utilization. Partial pressure decrease after physical exercise attenuates NOS activity in the NO-producing cells (Mantione et al., 2007). In humans, the exhaled NO level directly correlates with the exhaled oxygen level particularly in case of hypoxia (Verges et al., 2005). Moreover, there is a direct correlation between iNOS activity and oxygen concentration (Dweik et al., 1998). Reactive oxygen species play an important role in the pathophysiological processes in cardiovascular system (Faraci, 2006). It is interesting that H_2_O_2_ potentially mediates vascular adaptation during exercise (Sindler et al., 2009). Moreover, NO production by the capillary endothelium controls oxygen consumption by the mitochondria through chemical interaction between NO and iron-sulfur centers of the enzymes (Shen et al., 1995). Aerobic exercise increases eNOS expression at mRNA and protein levels in patients with CAD (Hambrecht et al., 2003). Similar results were obtained in pulmonary arteries from pigs (Johnson et al., 2001) and spontaneously hypertensive rats (Ma and Zhao, 2014). Jones and colleagues report that NOS inhibition by L-NAME significantly increases the absorption rate of pulmonary oxygen (VO_2_) in humans subjected to moderate cyclic exercise. In the beginning of moderate exercise, the internal inertia of oxidative metabolism can lead to the competitive inhibition of VO_2_ by NO in the mitochondrial cytochrome c oxidase. However, understanding of the detailed mechanisms of how L-NAME affects VO_2_ kinetics requires further studies (Jones et al., 2003).

Six-month exercise training reduces arterial pressure and is associated with an increase in nitrite/nitrate oxide contents in older women (Zaros et al., 2009). At the same time, when present in the blood stream, NO binds to hemoglobin that carries and metabolizes this molecule (Kosaka, 1999; Liao et al., 1999; Veeramachaneni et al., 1999; Gladwin et al., 2000; Taylor-Robinson, 2000). Progression of NO-hem complex formation *in vivo* is a rapidly reversible process (Gladwin et al., 2000; Taylor-Robinson, 2000). In the presence of low blood oxygenation, hemoglobin releases NO due to lower affinity to this molecule particularly in the venous blood (Kosaka, 1999; Taylor-Robinson, 2000).

## Mechanisms underlying exercise-induced NO production

In the presence of physical exercise, physical and chemical stimuli control NO production. In the endothelial cells, exercise stimulates NO synthesis through chemical mechanisms (Garcia, 2011). Chemical mechanisms involve interaction of endogenous/exogenous agonists (acetylcholine, bradykinin, and ATP) with the specific receptors on the endothelial cells. Evidence suggests that physical exercise stimulates release of these molecules. In exercise, the efferent nerve neuromuscular junctions are the physiological source of acetylcholine (Kingwell, 2000). Erythrocytes have been shown to release ATP in response both to low erythrocyte hemoglobin oxygen saturation (SO_2_) (Dietrich et al., 2000; Ellsworth et al., 2009) and to increased shear stress on the erythrocyte membrane (Wan et al., 2008). Interstitial bradykinin content is increased in the muscle contraction during strenuous exercise (Langberg et al., 2002). In the circumflex coronary artery, physical activity moderately increases the adenosine-stimulated NO production. A NOS inhibitor, L-NAME, moderately attenuates arteriolar dilatation in response to NOS activation in animals subjected to exercise (Thengchaisri et al., 2007).

According to data of Calvert and coworkers, β 3-adrenoceptors play critical role in the regulation of phosphorylation (activation) of eNOS and NO generation in response to exercise. Trained mice have the increased NO production and levels of nitrates and nitrosothiols in the heart (Calvert et al., 2011).

Physical impact on the vascular wall stimulates NO release in the blood vessels (Persson et al., 1993). The hypothetical physical stimulus affecting NO synthesis is the shear stress i.e., the friction force between fluid layers flowing at different speed. Human and animal studies suggest that exercise-induced cardiac output elevation contributes to the increased shear stress in the blood vessels (Persson et al., 1993). The increased exercise-induced shear stress, in turn, stimulates the release of vasorelaxation factor (NO) and augments eNOS and nNOS expression (Whyte and Laughlin, 2010).

**Figure 2:**
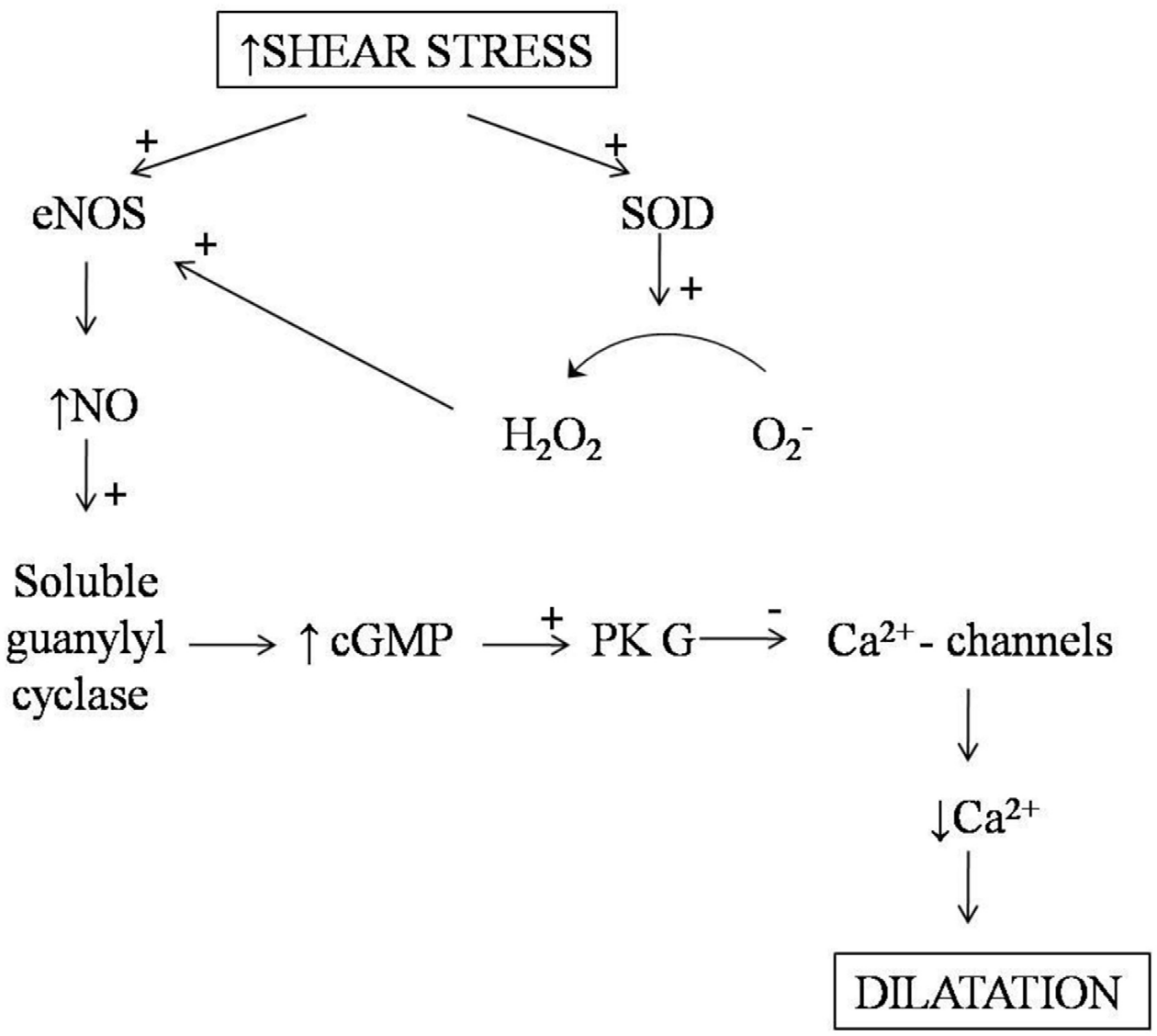
**NO-dependent pathways involved in shear stress-induced vascular dilatation**.

Mechanisms of the shear stress-induced NO synthesis are not completely understood. It is known that the endothelial cells express mechanoreceptors directly activating G-proteins, ion channels, and enzymes such as protein kinases and phosphatases generating second messengers (cGMP) (Zhan et al., 2003; Gielen et al., 2010). Blood flow delivers shear force to the vascular wall, causes deformation of the endothelial cells, and activates NO-cGMP-dependent signaling system (Barnes and Belvisi, 1993) (Figure 2). Vasodilation, triggered by shear stress, in the pulmonary blood vessels is less understood compared with that in the peripheral circulation. Pulmonary vascular reactions, associated with the changes in the exhaled NO content, remain under discussion (Sheel et al., 1999).

Shear stress stimulates the endothelium-dependent production of reactive oxygen species (ROS) that plays an important role in the cardiovascular system. Indeed, superoxide and hydrogen peroxide (H_2_O_2_) at low concentrations function as signaling molecules (Drouin et al., 2007; Larsen et al., 2008; Drouin and Thorin, 2009). Activation of eNOS can be caused by these ROS (Laurindo et al., 1994). Shear stress-induced NO production is accompanied by the expression of extracellular superoxide dismutase (SOD) catalyzing the rapid dismutation of superoxide into hydrogen peroxide and molecular oxygen (Gielen et al., 2010). Hydrogen peroxide then diffuses through the vascular wall and increases eNOS expression and activity (Drummond et al., 2000; Cai et al., 2001). Therefore, increased SOD1 and SOD3 expression potentiates the exercise-induced eNOS expression through hydrogen peroxide. On the other hand, eNOS expression is not elevated in catalase transgenic mice (Rush et al., 2003). Moreover, NO can directly interact with the mitochondrial ROS generated by NAD(P)H oxidase (NOX) and xanthine-oxidase (XO) (Gliemann et al., 2014).

## Significance

Evidence suggests that physical activity alters NO production in diseases. Nitric oxide protects pulmonary tissues in asthmatic patients during exercise. A bronchoprotective role of NO was initially demonstrated by the study with an inhalation of NOS inhibitor, NG-Methyl-L-arginine (Suman and Beck, 2002). In the pulmonary rehabilitation of patients with moderate chronic obstructive pulmonary disease, improved exercise tolerance can be attributed to an increase in the exhaled NO concentration. Exhaled NO content can represent a useful pathophysiological marker of adaptation to training in these patients (Clini et al., 2001).

Exercise protects the endothelium continuity through the increase in NO production (Xie et al., 2012). When individuals with preserved endothelial function are subjected to moderate/heavy exercise, L-arginine does not affect pulmonary vascular tone, but reverses the effects of NO-synthase inhibition (Green et al., 2011). Physical exercise structurally and functionally benefits the blood vessels and, in particularly, vascular endothelium both in healthy subjects and individuals with abnormal NO-induced vasorelaxation (Green et al., 2004). Exercise efficacy depends on training amount and effort/intensity. Short exercise rapidly increases biological activity of NO (Wei Xie et al., 2012) whereas prolonged training causes NO-dependent arterial remodeling and normalization of shear stress response (Maiorana et al., 2003). These, in turn, eliminate need for continuous functioning of NO-dependent systems to maintain vasodilation.

Physical activity significantly improves functioning of the cardiovascular system through the increase in NO bioavailability, potentiation of antioxidant defense, and decrease in the expression of ROS-forming enzymes (Rush et al., 2005). Regular exercise is a useful tool to fight cardiovascular diseases. Future studies should focus on identification of exercise approaches optimal for achieving the increased NO bioavailability and improved cardiovascular function (Gliemann et al., 2014).

## Conclusions

Existing data on exercise-mediated mechanisms of NO production in the cardiopulmonary system remain controversial. Nitric oxide is produced by various cell types including those present in the blood vessels. All these cells are potential sources of NO in the exhaled air. According to a vast pool of data, exercise controls NO synthesis through modulation of NOS activity (Laughlin et al., 2001; Boo and Jo, 2003; Gielen et al., 2005; Park et al., 2012). In response to exercise, the exhaled NO content increases, decreases, or remains unchanged depending on the presence of local factors. These factors comprise the levels NOS expression and activity, severity of oxidative stress, NO binding to antioxidant molecules hemoglobin and glutathione (Ricciardolo, 2003), and individual pattern of physical activity (Sessa et al., 1994; Laughlin et al., 2001). Evidence suggests the possible existence of the exercise amount/effort thresholds pivotal for the regulation of NO production. However, precise identification of these physical effort thresholds requires further studies (Garcia, 2011).

### Conflict of interest statement

The authors declare that the research was conducted in the absence of any commercial or financial relationships that could be construed as a potential conflict of interest.
